# Current state of trauma and violence in São Paulo - Brazil during the COVID-19 pandemic

**DOI:** 10.1590/0100-6991e-20202875

**Published:** 2021-02-05

**Authors:** MARCELO AUGUSTO FONTENELLE RIBEIRO-JUNIOR, PAOLA REZENDE NÉDER, SAMARA DE SOUZA AUGUSTO, YASMIN GARCIA BATISTA ELIAS, KAROLINE HLUCHAN, OTTO MAURO SANTO-ROSA

**Affiliations:** 1 - Pontifícia Universidade Católica de São Paulo PUCSP-Sorocaba, Disciplina de Cirurgia Geral e do Trauma - Sorocaba - SP - Brasil; 2 - Universidade Santo Amaro UNISA, São Paulo - SP - Brasil; 3 - Universidade Federal do Maranhão, Departamento de Cirurgia - São Luiz - MA - Brasil

**Keywords:** Pandemic, Coronavirus, Violence, Violence against Women, Wounds and Injuries, Pandemia, Coronavírus, Violência, Violência contra a Mulher, Ferimentos e Lesões

## Abstract

The coronavirus pandemic led society to adopt measures to contain its spread that generate impacts in the social, economic and psychological spheres, mainly due to social isolation. Some authors point out that social changes have generated changes in the various forms of trauma and violence. For this study, data collection for the years 2019 and 2020 was carried out on DATASUS - TABNET and on the website of the Secretariat of Public Security - SSP, considering various types of trauma and violence, with subsequent correlation analysis using the Kendall coefficient and correlation test. There was statistical significance, allowing a correlation with the negative pandemic for the rates of body injury due to traffic accidents, gunshot injuries, stab wounds, sexual violence, bodily injuries and interpersonal violence. As factors possibly associated with a reduction in the incidence of these variables, the literature presents some changes resulting from the pandemic, such as adherence to isolation, with a reduction in the flow of people on the street, and a decrease in reports of violence. The present study indicates that the findings may serve as a warning for future changes and for the adoption of preventive measures, however they represent the initial situation of the pandemic in São Paulo and, therefore, further investigations must be carried out with the course of the pandemic, which still remains.

## RESUMO

A pandemia do coronavírus levou a sociedade a adotar medidas de contenção de sua disseminação que geram impactos em âmbito social, econômico e psicológico, principalmente pelo isolamento social. Alguns autores apontam que as mudanças sociais geraram mudanças sobre as variadas formas de trauma e violência. Para este estudo, foi realizada coleta de dados referente aos anos de 2019 e 2020 no DATASUS - TABNET e no site da Secretaria de Segurança Pública- SSP, considerando diversos tipos de trauma e violência, com posterior análise de correlação pelo teste de coeficiente e correlação de Kendall. Houve significância estatística, permitindo realizar correlação com a pandemia de caráter negativo para as taxas de lesão corporal por acidente de trânsito, ferimento por arma de fogo, ferimento por arma branca, violência sexual, lesão corporal e violência interpessoal. Como fatores possivelmente associados a redução na incidência destas variáveis, a literatura apresenta algumas mudanças provenientes da pandemia, como adesão ao isolamento, com redução do fluxo de pessoas na rua, e diminuição de denúncias de violência. O presente estudo indica que os achados podem servir de alerta para mudanças futuras e para adoção de medidas preventivas, porém representam o quadro inicial da pandemia em São Paulo e, portanto, mais investigações devem ser elaboradas com o decorrer da pandemia, que ainda se mantém.

## INTRODUCTION

The new coronavirus, named SARS-CoV-2, belongs to the family Coronaviridae, being responsible for a severe acute respiratory syndrome (SARS-COV2) known as COVID-19. The first reported cases occurred in Wuhan, Hubei province, China, in December 2019. Disseminated to other continents, on March 11, 2020, the World Health Organization (WHO) declared a public health emergency of international interest, characterized as a pandemic^1^ ^-^ ^3^.

In the first half of October 2020, 38,789,204 cases and 1,095,097 deaths were confirmed worldwide, reported by WHO^4^. However, these numbers are considered to be lower than the reality due to the low availability of tests and monitoring in some countries^1^. On that same date, Brazil had 5,140,863 cases and 151,747 deaths, with the state of São Paulo being the most affected in the country^4^ ^-^ ^6^.

The severity of the pandemic is essentially due to the unavailability of vaccines and/or medications aimed at treating infected patients. Thus, the alternative recommended by the WHO for the containment of the virus is the isolation of suspected cases, social distance, hand hygiene and respiratory etiquette^1^ ^,^ ^7^. The measures, once implemented, can have severe repercussions in the social, economic and psychological impact of a society^8^ ^,^ ^9^.

In this context, some authors have already discussed the potential impacts of social changes during the COVID-19 pandemic on the various forms of trauma and violence^10^ ^-^ ^13^. In an analysis of the cities of Los Angeles and Indianapolis, a decline was identified in some types of crimes and an increase in others. Domestic violence was among those that increased during this period^10^. An Italian study has shown a decrease in the number of complaints of domestic violence, due to the close contact between victim and aggressor, the lack of opportunity for the victim to escape abuse and the reduction in contact with people beyond the residential environment^11^. Finlay et al. have analyzed the relationship between alcohol consumption and violence, and observed a 67% increase in sales in the UK during the pandemic, and the increase in calls to charities that work against domestic violence. In a study on firearm violence in New York, Chicago, Los Angeles and Baltimore, there has been a paradoxical trend of increasing these incidents during the COVID-19 pandemic^12^.

In Brazil, to date, there are no data correlating the numbers of trauma and violence after the establishment of social distancing due to the COVID-19 pandemic. The aim of the present study seeks to evaluate the characteristics of different types of trauma available in the official databases during the pandemic period in São Paulo, the largest city in South America, with more than 11 million inhabitants.

## MATERIALS AND METHODS

Data for analysis of the municipality of São Paulo were taken from the SUS computer department, DATASUS, (http://datasus.saude.gov.br), accessing the health information - TABNET, to have access to the Diseases and grievances for notification - SINAN. Data collection was carried out according to the month of the notifications and the type of violence: physical; sexual; rape; body strength / beating; stab and firearm, between the available periods of 2019 and 2020.

The other consulted database was the website of the Public Security Secretariat - SSP (https://www.ssp.sp.gov.br/), with statistical data being retrieved according to the occurrences recorded per month, knowing that the data of the SSP are updated quarterly. Out of the analyzed data, the types of violence were selected: intentional homicide; willful and wrongful homicide due to traffic accident; willful and guilty bodily injury; bodily injury caused by traffic accident; rape and femicide; in the 2019 and 2020 available periods.

Statistical analysis of the data allows inferring the dependence and association between the studied variables and the number of cases of COVID-19. However, this inference needs to be supported by a verification of the presence of dependence between the variables, whether such dependence is due to chance and, if any, whether it is a positive or negative association. The correlation analysis allows us to verify the dependence between the studied variables and the strength of the association, the closer to +1 the greater the positive association and the closer to -1 the greater the negative association. The Kendall coefficient and correlation was used, due to its greater precision to assess the correlation between two ordinal variables in small sample sizes.

The bibliographic search was carried out in scientific articles, using the terms “COVID-19 violence” and “COVID-19 trauma” in the PubMed, SciELO and Bireme databases.

## RESULTS

**Figure 1:**
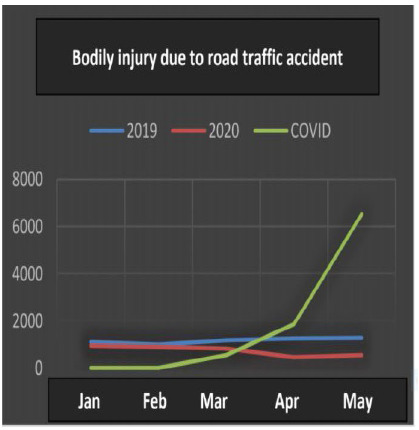
Bodily injury due to road traffic accident.

**Figure 2:**
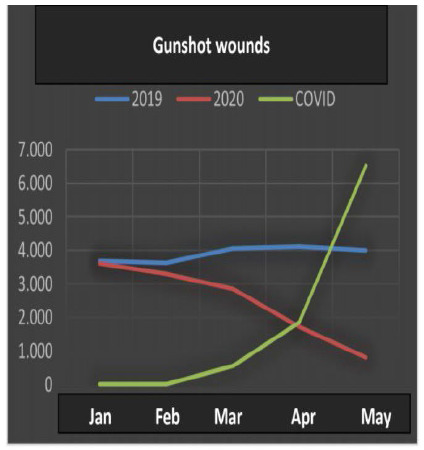
Gunshot wounds.

**Figure 3:**
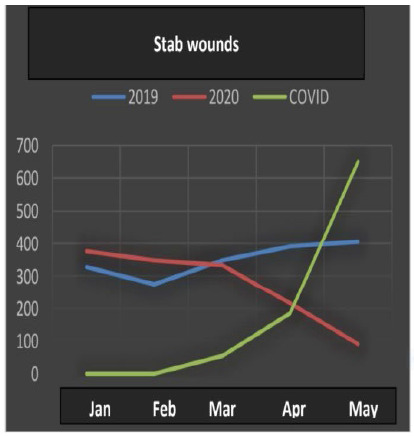
Stab wounds.

**Figure 4:**
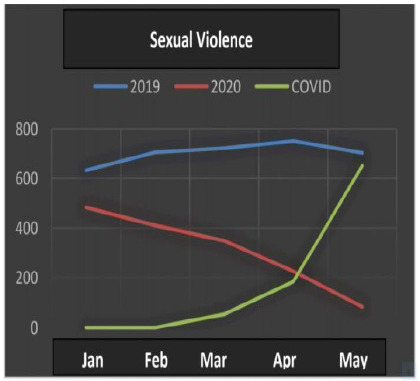
Sexual Violence.

**Figure 5:**
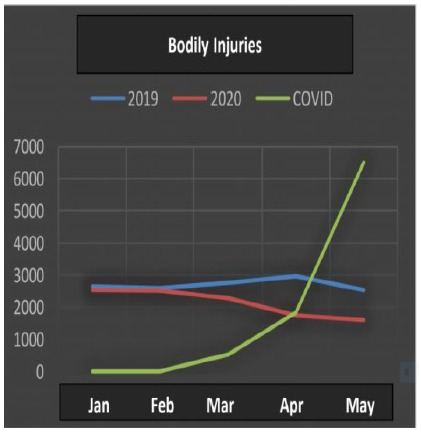
Bodily Injuries.

**Figure 6:**
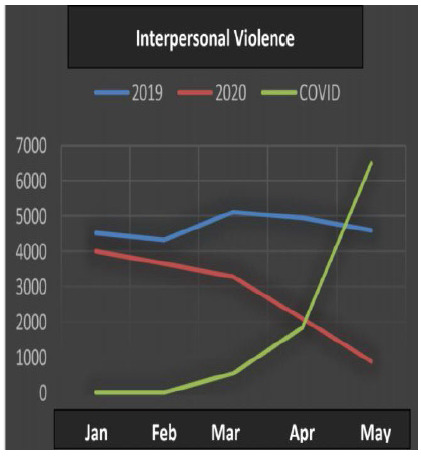
Interpersonal Violence.

All the data (Table 1) underwent statistical analysis, and the following types of violence achieved statistical significance (p <0.05): injuries due to road traffic accidents, firearm injuries, stab wounds, sexual violence, bodily injury and interpersonal violence. (Figures 1, 2, 3, 4, 5 and 6), which allows correlating the difference in the incidence of these types of violence between the years 2019 and 2020 with the number of COVID-19 cases. While homicide, femicide, rapes and homicide due to road traffic accidents presented had no statistical significant difference (p>0.05).

**Table 1:** Incidence of types of violence.

Violence	Month	2019	2020	p value	Kendall Test
Homicide	January	55	67	0.77	-0.242
	February	48	57		
	March	75	63		
	April	59	52		
	May	45	51		
Femicide	January	2	4	0.47	-0.241
	February	2	5		
	March	1	2		
	April	4	3		
	May	6	1		
Rape	January	214	238	0.08	-0.535
	February	186	205		
	March	238	219		
	April	229	128		
	May	199	120		
Homicide due to road traffic accidents	January	69	30	0.39	-0.267
	February	35	24		
	March	31	42		
	April	22	25		
	May	48	29		
Injuries due to traffic accidents	January	1106	950	0.02	-0.713
	February	988	900		
	March	1170	813		
	April	1255	453		
	May	1285	548		
Firearm injuries	January	3686	3607	0.01	-0.802
	February	3621	3280		
	March	4054	2847		
	April	4106	1712		
	May	3985	799		
Stab wound injuries	January	328	377	0.04	-0.624
	February	276	349		
	March	349	334		
	April	390	216		
	May	405	91		
Sexual violence	January	632	482	0.01	-0.802
	February	704	410		
	March	721	350		
	April	752	226		
	May	702	83		
Bodily injuries	January	2656	2538	0.01	-0.802
	February	2595	2517		
	March	2759	2272		
	April	2966	1740		
	May	2535	1600		
Interpersonal violence	January	4524	3993	0.01	-0.802
	February	4324	3643		
	March	5105	3277		
	April	4933	2100		
	May	4587	881		

The first cases of COVID-19 in the city of São Paulo occurred in March, totaling 5,389 cases, in that month. The subsequent months presented 18,438 and 65,038, respectively.

Regarding injuries due to road traffic accidents, there was a decrease in the number of cases (Kendall -0.713), showing a strong correlation with the incidence of COVID-19.

In reference to firearm injuries, a significant drop in the number of cases (Kendall -0.802) was observed in 2020, starting in March, increasing in April and May, showing a strong correlation with the pandemic.

Stab wounds also dropped in the number of cases (Kendall -0.624), mainly in May, which reinforces the strong correlation with COVID-19, in 2020.

Sexual violence, personal injury and interpersonal violence began the first half of 2020 in a decreasing curve (Kendall -0.802), with a further reduction in this rate with the increase in COVID-19 cases.

## DISCUSSION

The found results regarding the effects of social distance in the city of São Paulo had, for the most, adequate statistical significance for a reliable correlation with the scenario imposed by the pandemic. The analyzed signs of violence were homicide, femicide, rape, homicide by road traffic accidents, injuries due to road traffic accident, firearm injuries, stab wounds, sexual violence, bodily injury and interpersonal violence.

With respect to firearm and stab wounds, the results indicated negative associations after the emergence of the pandemic and its preventive measures of social distance. For firearms, the Kendall category value was -0.802, while for stab wounds it was -0.624. These results are different from those found in studies carried out in the cities of New York, Chicago, Baltimore and Los Angeles, at the beginning of the year 2020^13^. While our data reveal a significant reduction in the incidence of gunshot and stab wounds with the evolution of social isolation, the North American cities have shown an increase. As in the city of Santiago, Chile, an increase in penetrating trauma and a decrease in blunt traumatic injuries were assessed through a retrospective review. The latter authors also described the increased risk of death up to five times, when weapons are at home^14^. According to Sutherland et al., it is possible to explain this increase in some ways: 1) excessive time outside the workplace increases the chance to get involved in disagreements followed by consequent gunshot wounds; 2) with the increase in unemployment, low income and financial stress, there is a greater chance of being involved in crimes of theft with the use of weapons; 3) the increase in the consumption of alcoholic beverages was also another mentioned factor as a driver for violent attitudes. This analysis alone does not describe the period as a whole, requiring a greater assessment^13^. However, this variation in results in different countries leads us to reflect on preventive measures for problem secondary to the pandemic, which is the increase in violence related to emotional, psychological and financial damage in much of the society. While our data reveal an initial decrease, possibly due to the adherence to the social isolation, what such information leads us to think is that a change can be envisaged by the subsequent economic consequences, which will result in a future increase in violence, in general^13^.

In the cases of sexual violence, bodily injury and interpersonal violence, the results were different from those expected, considering the current literature. With greater coexistence in the domestic environment, it was expected that there would be an increase in these types of violence^8^ ^,^ ^9^ ^,^ ^15^ ^,^ ^16^. However, what was seen was a negative association for the three variables, the reduction by Kendall’s classification in - 0.802. According to Boserup et al., conditions associated with the quarantine period such as alcohol abuse, depression, and symptoms of post-traumatic stress can favor the environment for domestic violence^9^. In this type of violence, an individual usually has the control over the other, and it occurs in the forms of violence against the partner, the elderly and children, generally those who are most vulnerable within the domestic environment. This greater vulnerability is due to the fact that there is greater stress with domestic work as a result of the care for children, the elderly, and sick family members in the day-to-day life of individuals restricted from outside activities, with greater financial concerns and greater stress. The latter situations allow the abusers a greater space for manipulating their victims. As the contact with the others is reduced, as well as there is smaller community support network, many victims are discouraged from reporting their attackers^8^ ^,^ ^9^. According to Bradbury-Jones and Isham, social isolation has enabled abusers to dominate the means of communication and the victims’ interaction with the society, which makes it difficult to make the complaints^15^. This may explain our results, since, due to the greater home contact, less access to help from the community and health services, a significant increase in these values is expected. Unlike, in Chile, where there was an increase in domestic and interpersonal violence, in the period of social isolation^14^.

When analyzing bodily injuries from road traffic accidents, there was an important negative association, with Kendall of -0.713, over the months, also possibly related to the rate of adherence to social isolation. It is understood that with the reduction of the flow of people on the streets in their daily activities there is an expected reduction in automobile accidents^17^ ^,^ ^18^.

An observational Australian level I trauma center study revealed a decrease in the number of minor traumas such as falls and car crashes, from March to April of the current year, but there were no changes in admissions for major traumas, such as self-harm or robberies, and there were also no increases in data on domestic violence in that center in the studied period, corroborating some of the findings of the present study^17^. However, a descriptive study in the New Zealand Midland Trauma Registry revealed a significant reduction, from 43% of the overall admitted injuries during social isolation18. Other authors carried out an analysis of data on plastic surgery emergencies at a level I trauma center in Chicago, comparing data from three weeks before the quarantine and three weeks after, showing an increase in the percentage of injuries related to robberies and domestic violence, and a general decrease of patients who suffered collisions by motor vehicles^19^.

## CONCLUSION

The findings of the present study are compatible with the existing literature in relation to the reduction of car accident rates; however, our data had peculiarities in relation to sexual violence, bodily injury and interpersonal violence, as well as firearm and stab wounds, with negative correlations and contrary to the currently available literature.

It should be considered that the analyzed data represent the picture of trauma and violence in the early stages of the pandemic in São Paulo, so it is an analysis that must be carried on since the pandemic will continue for an indefinite period. Despite this, this initial portrait may be a warning for future changes and preventive measures, which can help control violence in the city of São Paulo and in other large cities around the world.
